# Supplementary material for: Implementation of guideline-directed medical treatment for ischemic heart disease management: A knowledge, attitude and practice based cross-sectional survey
Source: PLoS One. 2026 Feb 4;21(2):e0338634. doi: 10.1371/journal.pone.0338634 (PMC12872007; doi:10.1371/journal.pone.0338634)
Supplement: S1 File — (PDF) [file pone.0338634.s001.pdf]

S1 File: Knowledge, Attitudes, and Practices toward Guideline-Directed Medical Therapy in Ischemic Heart Disease (KAP-GDMT-IHD) Questionnaire

Demographic Information
• **Gender**<br>i. Male<br>ii. Female<br>• **Age in years**<br>o 22-33<br>o 34-44<br>o 45-60<br>• **Healthcare Professional**:<br>o Cardiologist<br>o Pharmacist<br>o General Physician<br>• **Years of Practice**:<br>o <1 years<br>o 1-5 years<br>o 6-10 years<br>• **Practice Setting**:<br>o Public hospital<br>o Private hospital/Clinic<br>• In your current role, how frequently do you engage in the management of IHD patients?<br>o Daily<br>o Weekly<br>o Occasionally<br>o Rarely
**Guideline-directed medical treatment (GDMT) for IHD**

1. **What are guideline-directed medical treatment (GDMT)?**
2. **What are the benefits of following GDMT?**
3. **What are some common guidelines used for ischemic heart disease (IHD)?**

   ☐ American college of cardiology (ACC) guidelines

   ☐ American heart association (AHA) guidelines

   ☐ European society of cardiology (ESC)

   ☐ Local/Institutional based guidelines

4. **How familiar are you with the latest guidelines for managing ischemic heart disease (e.g. American College of Cardiology (ACC)/American Heart Association (AHA) guidelines)?**

   ☐ Calcium channel blockers

   ☐ Somewhat familiar

   ☐ Not familiar at all

5. **Which of the following are key components of GDMT for IHD? (Select all that apply)**

   ☐ Antiplatelet therapy

   ☐ Beta-blockers

   ☐ ACE inhibitors or ARBs

   ☐ Statins

   ☐ Lifestyle modifications (diet, exercise)

   ☐ Others……………..

6. **Which of the following medications is NOT part of GDMT for IHD?**

- ☐ Beta-blockers
- ☐ ACE inhibitors/ARBs
- ☐ Proton pump inhibitors
- ☐ Statins

7. **What is the primary goal of GDMT in IHD?**
   - ☐ To reduce LDL cholesterol to target levels
   - ☐ To prevent progression of atherosclerosis and reduce cardiovascular events
   - ☐ To eliminate all cardiovascular risk factors
   - ☐ To improve exercise tolerance only

8. **Which of the following is a first-line antiplatelet therapy recommended for IHD patients?**
   - ☐ Clopidogrel
   - ☐ Aspirin
   - ☐ Warfarin
   - ☐ Ticagrelor

9. **Which patient population benefits most from high-intensity statin therapy?**
   - ☐ Patients with LDL < 100 mg/dL
   - ☐ Patients with diabetes and established IHD
   - ☐ Patients with normal lipid profiles
   - ☐ Patients over 70 years of age

10. **In IHD patients with reduced ejection fraction, which GDMT drug class is recommended?**

- ☐ Beta-blockers
- ☐ Calcium channel blockers
- ☐ Loop diuretics
- ☐ Nitrates

11. **Are you aware of recent updates to GDMT in IHD management in the last 2 years?**

- ☐ Yes
- ☐ No

Attitudes (A)

1. **GDMT is essential for improving patient outcomes in IHD.**
   - o Strongly agree
   - o Agree
   - o Neutral
   - o Disagree
   - o Strongly disagree

2. **In your opinion, is it to implement GDMT in routine clinical practice for IHD patients?**
   - o Strongly agree
   - o Agree
   - o Neutral
   - o Disagree
   - o Strongly disagree

3. **Do you believe that pharmacist involvement in the care of IHD patients improves adherence to GDMT?**
   - o Strongly agree
   - o Agree

- Neutral
- Disagree
- Strongly disagree

4. **Are you in the current multidisciplinary team approach to managing IHD, including cardiologists and pharmacists?**
    - Strongly agree
    - Agree
    - Neutral
    - Disagree
    - Strongly disagree

5. **Following GDMT guidelines can significantly reduce hospitalizations in IHD patients.**
    - Strongly agree
    - Agree
    - Neutral
    - Disagree
    - Strongly disagree

6. **What barriers, if any, do you perceive in implementing GDMT in your practice? (Select all that apply)**
    - Lack of patient adherence
    - Limited time in consultations
    - Complexity of guidelines
    - Insufficient support from other healthcare professionals
    - Cost of therapies

Other: ____________

7. **How often do you consult pharmacists when managing IHD patients?**
    - Always
    - Often
    - Sometimes
    - Rarely
    - Never

8. Healthcare professionals in my workplace have adequate training on GDMT.
    - Strongly agree
    - Agree
    - Neutral

- Disagree
- Strongly disagree

9. I feel confident in prescribing or recommending GDMT for IHD patients

- Strongly agree
- Agree
- Neutral
- Disagree
- Strongly disagree

10. . Implementing GDMT is challenging due to patient non-adherence.

- Strongly agree
- Agree
- Neutral
- Disagree
- Strongly disagree

Practices (P)

1. **How frequently do you follow GDMT when managing patients with ischemic heart disease?**
   - Always
   - Often
   - Sometimes
   - Rarely
   - Never

2. **How frequently do you counsel patients on GDMT and lifestyle modifications?**
   - Always
   - Often
   - Sometimes
   - Rarely
   - Never

3. **When was the last time you reviewed the GDMT guidelines for IHD?**
   - Within the last 6 months
   - 6-12 months ago
   - More than 1 year ago
   - Never reviewed

4. **In your practice, how often do you adjust a patient's medication regimen based on the GDMT guidelines for IHD?**
    - Always
    - Often
    - Sometimes
    - Rarely
    - Never

5. **Do you regularly involve pharmacists in decision-making for IHD patient treatment plans?**
    - Yes
    - No
    - Sometimes

6. **What steps do you take to improve adherence to GDMT in your IHD patients? (Select all that apply)**
    - Educating patients about the benefits of therapy
    - Collaborating with pharmacists for medication counseling
    - Monitoring lab values regularly
    - Using reminder systems for follow-ups
    - Other: ___________

7. **Which of the following methods do you use to assess patient adherence to GDMT?**
    - Direct questioning during follow-up
    - Pill count
    - Pharmacy refill data
    - Medication adherence apps/tools
    - Other: ___________

8. **How often do you prescribe DAPT for patients following percutaneous coronary intervention (PCI)?**
    - Always
    - Often
    - Sometimes
    - Rarely
    - Never

9. **In your practice, what percentage of your IHD patients are on optimal GDMT?**
    - 0-25%

- 26-50%
- 51-75%
- 76-100%

10. **How frequently do you reassess a patient's GDMT based on changes in their clinical condition?**
    - Every follow-up visit
    - Every 6 months
    - Annually
    - Only when symptoms worsen
    - Never
