# Supplementary material for: Implementation of guideline-directed medical treatment for ischemic heart disease management: A knowledge, attitude and practice based cross-sectional survey
Source: PLoS One. 2026 Feb 4;21(2):e0338634. doi: 10.1371/journal.pone.0338634 (PMC12872007; doi:10.1371/journal.pone.0338634)
Supplement: S1 Table — The correct answer is presented in percentage. (DOCX) [file pone.0338634.s003.docx]

**S1 Table: Summary of questions for Knowledge, Attitudes and Practices towards GDMT. The correct answer is presented in percentage**

| **Domain** | **Question items** | **Correct/ Preferred response** |
| --- | --- | --- |
| **Knowledge** | Q1: Are you familiar with the current clinical guidelines for the management of IHD (e.g., ACC/AHA guidelines)? | (80.25%) |
|  | Q2: Are you familiar with key components (e.g. antiplatelet therapy, beta blockers, ACE inhibitors or ARBs, statins and lifestyle modifications) of GDMT? | 55.2% |
|  | Q3: Are you aware of recent updates to GDMT in IHD management in the last 2 years? | 22.7% |
|  | Q4: Are you aware of the specific role of clinical pharmacists in implementing GDMT? | 56.2% |
| **Attitude** | A1: Do you think adhering to GDMT is IMPORTANT for improving patient outcomes in IHD? | 44.6% |
|  | A2: is it feasible to implement GDMT in routine clinical practice for IHD patients? | 91.5% |
|  | A3: Do you believe that pharmacist involvement in the care of IHD patients improves adherence to GDMT? | 78.2% |
|  | A4: Are you confident in the current multidisciplinary team approach to managing IHD, including cardiologists and pharmacists? | 60.1% |
|  | What barriers, if any, do you perceive in implementing GDMT in your practice? (Select all that apply)   - Lack of patient adherence - Limited time in consultations - Complexity of guidelines - Insufficient support from other healthcare professionals | 25%  47.4%  15.8%  11.8% |
| **Practice** | P1: Do you believe that having a clinical pharmacist involved enhances patient education and medication adherence? | 92.5% |
|  | P2: In your practice, do you often adjust a patient’s medication regimen based on the GDMT for IHD? | 74.01% |
|  | P3: Do you regularly involve pharmacists in decision-making for IHD patient treatment plans? | 34.2% |
|  | P4: Do you frequently reassess a patient’s GDMT based on changes in their clinical condition? | 55.1% |
|  | P5: How frequently do you reassess a patient’s GDMT based on changes in their clinical condition?  Every 6 months  Annually  Only when symptoms worsen  Never | (16.1%)  (22.4%)  (45%)  (16.5%) |
|  | P6: Which of the following methods do you use to assess patient adherence to GDMT?   - - Direct questioning during follow-up   - Pill count   - Pharmacy refill data   - Medication adherence apps/tools | (56.5%)  (10.2%)  (22.7%)  (10.6%) |
