# Supplementary material for: Implementation of guideline-directed medical treatment for ischemic heart disease management: A knowledge, attitude and practice based cross-sectional survey
Source: PLoS One. 2026 Feb 4;21(2):e0338634. doi: 10.1371/journal.pone.0338634 (PMC12872007; doi:10.1371/journal.pone.0338634)
Supplement: S2 Table — (DOCX) [file pone.0338634.s004.docx]

**S2 Table: Descriptive statistics of KAP**

| **KAP** | **N** | **Minimum** | **Maximum** | **Mean** | **Std. Deviation** |
| --- | --- | --- | --- | --- | --- |
| Knowledge | 76 | 13.00 | 22.00 | 18.6447 | 2.02454 |
| Attitude | 76 | 7.00 | 14.00 | 10.4211 | 2.06729 |
| Practice | 76 | 7.00 | 14.00 | 9.5132 | 2.55339 |
| Total | 76 |  |  |  |  |
