# Supplementary material for: Implementation of guideline-directed medical treatment for ischemic heart disease management: A knowledge, attitude and practice based cross-sectional survey
Source: PLoS One. 2026 Feb 4;21(2):e0338634. doi: 10.1371/journal.pone.0338634 (PMC12872007; doi:10.1371/journal.pone.0338634)
Supplement: S3 Table — (DOCX) [file pone.0338634.s005.docx]

**S3 Table: Correlation of HCPs vs KAP scores**

| **Variables** | | **R value** | **df** | **P value** |
| --- | --- | --- | --- | --- |
| HCPs | Knowledge | –0.499 ** | 74 | < 0.001 |
| HCPs | Attitude | –0.262 * | 74 | 0.022 |
| HCPs | Practice | –0.088 | 74 | 0.449 |
| **Note**: r = Pearson correlation (point-biserial); p < 0.05 = significant (*); p < 0.01 = highly significant (**).  HCPs coded as: cardiologist = 1, pharmacist = 2. | | | | |
