# Supplementary material for: Implementation of guideline-directed medical treatment for ischemic heart disease management: A knowledge, attitude and practice based cross-sectional survey
Source: PLoS One. 2026 Feb 4;21(2):e0338634. doi: 10.1371/journal.pone.0338634 (PMC12872007; doi:10.1371/journal.pone.0338634)
Supplement: S4 Table — (DOCX) [file pone.0338634.s006.docx]

**S4 Table: Multiple Linear Regression Analysis Predicting KAP Toward GDMT Among Cardiologists and Pharmacists**

| **KAP Model** | **R** | **R Square** | **Adjusted R Square** | **Std. Error of the Estimate** | **Change Statistics** | | | | |
| --- | --- | --- | --- | --- | --- | --- | --- | --- | --- |
|  |  |  |  |  | **R Square Change** | **F Change** | **df1** | **df2** | **Sig. F Change** |
| **Knowledge** | 0.680^a^ | 0.463 | 0.416 | 1.54729 | 0.463 | 9.90 | 6 | 69 | **0.000** |
| **Attitude** | 0.530^a^ | 0.281 | 0.218 | 1.82782 | 0.287 | 4.49 | 6 | 69 | **0.001** |
| **Practice** | 0.152^a^ | 0.023 | -0.062 | 2.63 | 0.023 | 0.27 | 6 | 69 | 0.948 |
