# Supplementary material for: Implementation of guideline-directed medical treatment for ischemic heart disease management: A knowledge, attitude and practice based cross-sectional survey
Source: PLoS One. 2026 Feb 4;21(2):e0338634. doi: 10.1371/journal.pone.0338634 (PMC12872007; doi:10.1371/journal.pone.0338634)
Supplement: Attachment — Submitted filename: PONE-D-25-18404_reviewer (1).pdf [file pone.0338634.s007.pdf]

PLOS ONE

Implementation of Guideline-Directed Medical Treatment for Ischemic Heart Disease Management: A Knowledge, Attitude and Practice Based Cross-Sectional Survey

--Manuscript Draft--

**Manuscript Number:**	PONE-D-25-18404
**Article Type:**	Research Article
**Full Title:**	Implementation of Guideline-Directed Medical Treatment for Ischemic Heart Disease Management: A Knowledge, Attitude and Practice Based Cross-Sectional Survey
**Short Title:**	Guideline-Directed Medical Treatment for Ischemic Heart Disease Management
**Corresponding Author:**	Amjad Khan, Ph.D.<br>Quaid-i-Azam University<br>Islamabad, PAKISTAN
**Keywords:**	Ischemic heart disease; Guideline-directed medical treatment; Knowledge; attitude and Practice; Healthcare professionals; Interdisciplinary collaboration
**Abstract:**	Background<br><br>The effective management of ischemic heart disease relies profoundly on guideline-directed medical treatment, but compliance remains challenging due to gaps in knowledge, attitudes, and practices among healthcare professionals.<br><br>Aim<br><br>The study aimed to identify the knowledge, attitudes, and practices concerning guideline-directed medical treatment for ischemic heart disease management amongst healthcare professionals (HCPs) to elucidate their respective roles, challenges, and facilitators in providing optimal care to patients.<br><br>Methods<br><br>A cross-sectional survey of HCPs including, cardiologists and pharmacists was conducted from 5th July to 9th September, 2024, to assess their knowledge of GDMT, perspectives on its use, and clinical practices. Data was collected from Punjab Institute of Cardiology, Lahore, Pakistan using convenience sampling through a validated questionnaire. The relationships between KAP-based factors and demographic traits were evaluated using an independent sample t-test, pearson correlation analysis, and multiple linear regression. Statistical adjustment for multiple comparison was done by Bonferroni correction<br><br>Results<br><br>Seventy-six study participants completed the survey questionnaire. In the final sample, 42 participants (55.3%) were cardiologists, while 34 participants (44.7%) were pharmacists. The average knowledge score was 3.210 (SD: 0.498, range: 0–4). The understanding of current clinical recommendations for the care of IHD was highest (80.25%), whereas awareness of recent updates to GDMT in IHD management over the past two years was lowest (22.7%). The average score of practice was 5.921 (SD: 1.05, range: 0 to 8). Moreover, the results of the multiple linear regression analysis of variables showed that HCPs had significantly different levels of knowledge regarding the application of GDMT (β: 0.051, P> 0.005).<br><br>Conclusion<br><br>This study found that HCPs had a moderate knowledge, high understanding of current clinical guidelines and low practice score regarding implementation of GDMT in healthcare settings. Improved adherence to GDMT through augmented interdisciplinary cooperation could play a crucial role in optimizing care for IHD patients.

Powered by Editorial Manager® and ProduXion Manager® from Aries Systems Corporation

**Order of Authors:**	Umm-e- Kalsoom, MPhil
	Syed Sikandar Shah, PhD
	Amjad Khan, Ph.D.
	Ayesha Iqbal, PhD
	Syed Mansoor Ahmed, MPhil
	Yu Fang, PhD
**Additional Information:**	
**Question**	**Response**
**Financial Disclosure**<br><br>Enter a financial disclosure statement that describes the sources of funding for the work included in this submission. Review the submission guidelines for detailed requirements. View published research articles from *PLOS ONE* for specific examples.<br><br>This statement is required for submission and **will appear in the published article** if the submission is accepted. Please make sure it is accurate.<br><br>**Funded studies**<br>Enter a statement with the following details:<br>• Initials of the authors who received each award<br>• Grant numbers awarded to each author<br>• The full name of each funder<br>• URL of each funder website<br>• Did the sponsors or funders play any role in the study design, data collection and analysis, decision to publish, or preparation of the manuscript?<br><br>Did you receive funding for this work?	The author(s) received no specific funding for this work.
**Competing Interests**<br><br>Use the instructions below to enter a competing interest statement for this submission. On behalf of all authors, disclose any competing interests that could be perceived to bias this work—acknowledging all financial support and any other relevant financial or non-financial competing interests.	No authors have competing interest

This statement is required for submission and **will appear in the published article** if the submission is accepted. Please make sure it is accurate and that any funding sources listed in your Funding Information later in the submission form are also declared in your Financial Disclosure statement.<br><br>View published research articles from *PLOS ONE* for specific examples.<br><br>**NO authors have competing interests**<br><br>Enter: *The authors have declared that no competing interests exist.*<br><br>**Authors with competing interests**<br><br>Enter competing interest details beginning with this statement:<br><br>*I have read the journal's policy and the authors of this manuscript have the following competing interests: [insert competing interests here]*<br><br>* typeset	
**Ethics Statement**<br><br>Enter an ethics statement for this submission. This statement is required if the study involved:<br><br>• Human participants<br>• Human specimens or tissue<br>• Vertebrate animals or cephalopods<br>• Vertebrate embryos or tissues<br>• Field research<br><br>Write "N/A" if the submission does not require an ethics statement.<br><br>General guidance is provided below. Consult the submission guidelines for detailed instructions. **Make sure that all**	The bioethics committee of faculty of biological sciences, Quaid-i-Azam University, Islamabad (Ref. #: BEC-FBS-QAU2021-270) and the ethical review committee of Punjab Institute of Cardiology Hospital, Lahore (Ref. #: RTPGME-Research-204) had reviewed and approved this study in terms of its scientific substance and conformity with all relevant research and human subject regulations. Written inform consent from the study participants were taken before their enrollment in the study.

Powered by Editorial Manager® and ProduXion Manager® from Aries Systems Corporation

**information entered here is included in the Methods section of the manuscript.**<br><br>**Format for specific study types**<br><br>**Human Subject Research (involving human participants and/or tissue)**<br>• Give the name of the institutional review board or ethics committee that approved the study<br>• Include the approval number and/or a statement indicating approval of this research<br>• Indicate the form of consent obtained (written/oral) or the reason that consent was not obtained (e.g. the data were analyzed anonymously)<br><br>**Animal Research (involving vertebrate animals, embryos or tissues)**<br>• Provide the name of the Institutional Animal Care and Use Committee (IACUC) or other relevant ethics board that reviewed the study protocol, and indicate whether they approved this research or granted a formal waiver of ethical approval<br>• Include an approval number if one was obtained<br>• If the study involved *non-human primates*, add *additional details* about animal welfare and steps taken to ameliorate suffering<br>• If anesthesia, euthanasia, or any kind of animal sacrifice is part of the study, include briefly which substances and/or methods were applied<br><br>**Field Research**<br><br>Include the following details if this study involves the collection of plant, animal, or other materials from a natural setting:<br>• Field permit number<br>• Name of the institution or relevant body that granted permission	
**Data Availability**<br><br>Authors are required to make all data underlying the findings described fully available, without restriction, and from the time of publication. PLOS allows rare exceptions to address legal and ethical	No - some restrictions will apply

concerns. See the PLOS Data Policy and FAQ for detailed information.<br><br>A Data Availability Statement describing where the data can be found is required at submission. Your answers to this question constitute the Data Availability Statement and **will be published in the article**, if accepted.<br><br>**Important:** Stating 'data available on request from the author' is not sufficient. If your data are only available upon request, select 'No' for the first question and explain your exceptional situation in the text box.<br><br>Do the authors confirm that all data underlying the findings described in their manuscript are fully available without restriction?	
**Describe where the data may be found in full sentences. If you are copying our sample text, replace any instances of XXX with the appropriate details.**<br><br>• If the data are **held or will be held in a public repository**, include URLs, accession numbers or DOIs. If this information will only be available after acceptance, indicate this by ticking the box below. For example: *All XXX files are available from the XXX database (accession number(s) XXX, XXX.*).<br>• If the data are all contained **within the manuscript and/or Supporting Information files**, enter the following: *All relevant data are within the manuscript and its Supporting Information files.*<br>• If neither of these applies but you are able to provide **details of access elsewhere**, with or without limitations, please do so. For example:<br><br>*Data cannot be shared publicly because of [XXX]. Data are available from the XXX Institutional Data Access / Ethics Committee (contact via XXX) for researchers who meet the criteria for access to confidential data.*	Data cannot be shared publicly because of researchers who meet the criteria for access to confidential data.

The data underlying the results presented in the study are available from (include the name of the third party and contact information or URL).<br>• This text is appropriate if the data are owned by a third party and authors do not have permission to share the data.<br><br>* typeset	
Additional data availability information:	

Manuscript

Click here to access/download;Manuscript;Updtaed Manuscript file -PLOS one.docx

Implementation of Guideline-Directed Medical Treatment for Ischemic Heart Disease Management: A Knowledge, Attitude and Practice Based Cross-Sectional Survey

Umm-e-Kalsoom, MPhil[1, 2], Syed Sikandar Shah[3], Amjad Khan, PhD* [1, 4, 5], Ayesha Iqbal, PhD [6, 7], Syed Mansoor Ahmed, MPhil[8], Yu Fang, PhD [4, 5]

[1]Department of Pharmacy, Quaid-i-Azam University, Islamabad, Pakistan

[2]Department of Practice & Policy, School of Pharmacy, University College London, London, UK

[3]Department of Clinical Pharmacy and Pharmacology, RAK College of Pharmacy, RAK Medical & Health Sciences University, Ras Al Khaimah, UAE

[4]Department of Pharmacy, The First Affiliated Hospital, Xi'an Jiaotong University, Xi'an, China

[5]Department of Pharmacy Administration and Clinical Pharmacy, School of Pharmacy, Health Science center, Xi'an Jiaotong University, Xi'an, China

[6]Department of Pharmacy Practice and Policy, University Park Campus, University of Nottingham, Nottingham, NG72RD

[7]Faculty of Medicine and Dentistry, University of Alberta, Canada, AB T6G1C9

[8]Department of Biotechnology, Mirpur University of Science and Technology, Mirpur Azad Kashmir

E-mail Addresses:

ukalsoom@bs.qau.edu.pk, syed.sikandar@rakmhsu.ae, amjadkhan@qau.edu.pk, aiqbal6@ualberta.ca, ,mansoor_shah125@yahoo.com, yufang@mail.xjtu.edu.cn

Corresponding authors:

Dr. Amjad Khan (Email: amjadkhan@qau.edu.pk)

Abstract

Background

The effective management of ischemic heart disease relies profoundly on guideline-directed medical treatment, but compliance remains challenging due to gaps in knowledge, attitudes, and practices among healthcare professionals.

Aim

The study aimed to identify the knowledge, attitudes, and practices concerning guideline-directed medical treatment for ischemic heart disease management amongst healthcare professionals (HCPs) to elucidate their respective roles, challenges, and facilitators in providing optimal care to patients.

Methods

A cross-sectional survey of HCPs including, cardiologists and pharmacists was conducted from 5th July to 9th September, 2024, to assess their knowledge of GDMT, perspectives on its use, and clinical practices. Data was collected from Punjab Institute of Cardiology, Lahore, Pakistan using convenience sampling through a validated questionnaire. The relationships between KAP-based factors and demographic traits were evaluated using an independent sample t-test, pearson correlation analysis, and multiple linear regression. Statistical adjustment for multiple comparison was done by Bonferroni correction

Results

Seventy-six study participants completed the survey questionnaire. In the final sample, 42 participants (55.3%) were cardiologists, while 34 participants (44.7%) were pharmacists. The average knowledge score was 3.210 (SD: 0.498, range: 0–4). The understanding of current clinical recommendations for the care of IHD was highest (80.25%), whereas awareness of

recent updates to GDMT in IHD management over the past two years was lowest (22.7%). The average score of practice was 5.921 (SD: 1.05, range: 0 to 8). Moreover, the results of the multiple linear regression analysis of variables showed that HCPs had significantly different levels of knowledge regarding the application of GDMT (β: 0.051, $P > 0.005$).

Conclusion

This study found that HCPs had a moderate knowledge, high understanding of current clinical guidelines and low practice score regarding implementation of GDMT in healthcare settings. Improved adherence to GDMT through augmented interdisciplinary cooperation could play a crucial role in optimizing care for IHD patients.

Key words

Ischemic heart disease, Guideline-directed medical treatment, Knowledge, attitude and Practice, Healthcare professionals, Interdisciplinary collaboration

Highlights

The following practice implications can be inferred from the Knowledge, Attitude, and Practice (KAP) survey results about the application of Guideline-Directed Medical Therapy (GDMT) in the management of Ischemic Heart Disease (IHD):

- The KAP survey identifies gaps in research among HCPs in implementation of GDMT for IHD management.
- The findings also identify barriers in implementation of GDMT in practice.
- The low practice score highlights the need for collaborative care models, in which primary care physicians, cardiologists, and clinical pharmacists collaborate to maximize GDMT adherence.

Introduction

Ischemic heart disease (IHD) is the most common cause of deaths among cardiovascular diseases (CVDs) and a significant global health risk [1,2]. Its prevalence is rapidly increasing, with a predicted increase to 1,845 per 100,000 by 2030 [3]. Guidelines-directed medical treatment (GDMT) provides a comprehensive strategy for managing ischemic heart disease, incorporating lifestyle modifications and evidence-based pharmacological interventions [4-6]. GDMT is a cornerstone of IHD treatment, encompassing a range of pharmacological strategies aimed at decelerating atherosclerosis progression, preventing coronary thrombosis, and improving patients' overall well-being [7]. A key component of GDMT, clinical pharmacists provide a significant advantage in offering comprehensive drug management, ensuring adherence to evidence-based protocols, and optimizing pharmacotherapy [8]. As members of a multidisciplinary healthcare team, clinical pharmacists are important in enhancing GDMT effectiveness [5]. Previous studies have demonstrated that the efficacy of GDMT correlates with improved outcomes for patients experiencing acute ischemic heart failure, underscoring the critical importance of pharmacological interventions as the primary treatment approach [9]. Furthermore, Mohiuddin et al. also reported that pharmacists are working as important part of healthcare team as medical counselors, educators and advocates in addition to dispensing medications, and are considered among the most trusted and accessible health care professionals [10].

However, there are substantial gaps in the GDMT dose initiation and titration among patients despite strong evidence and recommendations from organizations such as the American Heart Association (AHA), European Society of Cardiology (ESC), and American College of Cardiology (ACC) [11]. Interestingly, a Delphi et al. consensus among Belgian cardiologists emphasized the importance of swift implementation of four cornerstone medical therapies in newly diagnosed HFrEF patients, individualizing treatment sequencing, and ensuring regular

follow-up to optimize therapy, and highlighting the evolving nature of GDMT implementation [12]. Implementation of GDMT remains suboptimal, with minimal improvement even after heart failure hospitalization [13]. In order to bridging gaps and optimizing implementation of GDMT for heart failure among healthcare professionals, previous literature reported various implementation strategies such as rapid initiation of GDMT in hospital, improvement in patient medication adherence, addressing clinical inactivity, engagement in quality improvement registries, improving affordability establishing multidisciplinary clinics, and implementing interventions in the recent decade [14].

The Knowledge, Attitudes, and Practices (KAP) framework provides essential insights into healthcare professionals' comprehension, beliefs, and actions regarding guideline implementation. Understanding how healthcare professionals perceive, implement, and adhere to GDMT for IHD patients is crucial for identifying barriers and facilitators in clinical environments. This study seeks to investigate the knowledge, attitudes, and practices of healthcare team including pharmacists and cardiologists, regarding the implementation of GDMT in healthcare settings. The effective implementation of GDMT will depend on the knowledge and practices of cardiologists and pharmacists working under comparable circumstances. It is important to analyze how similar metrics may be interpreted differently across varied situations, especially within an underdeveloped healthcare system.

Methods

Study design and settings

A cross-sectional survey of healthcare professionals was carried out from 5 July to 9 September 2024 to assess the knowledge, attitudes, and practices of GDMT implementation. The research adopted a questionnaire-based methodology and distributed questionnaires to participants working at the Punjab Institute of Cardiology (PIC), Lahore, Pakistan. The PIC is a 547-beds tertiary care hospital and is the first heart institute in the province of Lahore, Pakistan. It offers

excellent treatment for all heart conditions and receives patients from all over Pakistan. The PIC has a total of 147 cardiologists and 35 pharmacists along with nurses and other paramedical staff working in various departments. This is the only tertiary care hospital in Lahore, Pakistan that functions as a public facility in the morning and a private institution in the evening.

Study participants and sampling

Inclusion Criteria

The respondents to this survey were healthcare professionals, including registered cardiologists and pharmacists, aged 22 years or older, working in PIC, Lahore. Medical officers directly involved in the management of IHD were also included in this study.

Exclusion Criteria

Nurses and other paramedical staff working in PIC who were directly involved in the management of IHD were excluded from the study. Cardiologists and pharmacists aged less than 22 years who had not completed their graduation degree and were not involved in medical care for IHD patients were also excluded from the study. HCPs who had recently graduated and retired were not included in this study. Participants who refused to participate in this study were also excluded.

Sampling

This study was conducted in a tertiary-care PIC hospital with a limited number of specialized pharmacists and cardiologists involved in IHD management. All participants provided voluntary verbal and written informed consent prior to completing the questionnaire. A convenience sampling strategy was employed to gather data and ensure a wide representation of the study participants. Due to the nature of this study, the sample size was not determined to maximize responses.

Questionnaire

The questionnaire was developed and validated through an extensive literature review and approval from both supervisors and independent researchers in the relevant subject. The expert team reviewed each item of the questionnaire for clarity, relevance, and comprehensiveness using the Content Validity Index (CVI), retaining items that met the minimum criterion of 0.78 [15]. The questionnaire (refer to Supplementary File 1) was divided into four sections. The preliminary section contained data on respondents' demographics. The second section consisted of 11 questions, including those that evaluated HCPs knowledge and understanding of GDMT for the management of IHD. The subsequent section consisted of 10 questions aimed at evaluating the perspectives on guideline-directed medical therapy for IHD. Finally, the practice-based questionnaires consisted of 10 questions to evaluate their behaviour and adherence to the implementation of GDMT in the management of IHD. The questionnaire was available in English and required approximately 15 minutes to complete. The research team assessed the practicality, readability, and internal consistency of the questionnaire with a small participant group of 20–30 HCPs through pilot testing. The Cronbach's alpha value (0.712) also confirmed internal reliability.

The most frequently used Bloom's cutoff points were used for KAP assessment: 80-100% (excellent KAP), 60–79% (moderate KAP), and less than 60% (bad KAP) [16-18]. The responses were scored for determination of knowledge, while the Likert scale was used for attitude, and frequency-based responses were used for perception calculation [19].

Statistical analysis

Survey responses were obtained, and the answers were evaluated and scored based on the established criteria. A descriptive analysis was performed on the demographic data. Additionally, an independent sample t-test, one-way analysis of variance (ANOVA), and

multiple linear regression were employed to investigate the relationship between sociodemographic variables and KAP-based characteristics. Pearson's correlation analysis was used to assess the associations among variables. All statistical analyses were conducted utilizing IBM SPSS 21 for Windows was used for statistical analysis. To control the overall rate of false positive results, statistical adjustment for multiple comparisons was performed using Bonferroni correction (value is 0.0125).

Results

Seventy-six study participants completed the survey questionnaire. In the final sample, 42 participants (55.3%) were cardiologists, while 34 participants (44.7%) were pharmacists. Out of 76 study participants, 41 (53.9%) were female, 39 (51.9%) were aged between 28 and 33 years, 43 (56.6%) had attained post-graduate degrees or higher, 46 (60.5%) were employed in public hospitals, and 37 (44.7%) had 1-5 years of clinical experience. The sociodemographic variables and knowledge score are presented in Table 1.

Table 1: sociodemographic characteristics and the GDMT knowledge score

Variables	Categories	N (%)	Knowledge score (mean ± standard deviation)	P value
Gender	Male	35 (46.1)	3.20 ± 0.473	0.448
	Female	41 (53.9)	3.21 ± 0.525	
Age in year	22-27	22 (28.9)	3.00 ± 0.00	0.000
	28-33	39 (51.3)	3.307 ± 0.569	
	34-39	15 (19.7)	3.266 ± 0.593	
Educational Level	Graduation	33 (43.4)	3.030 ± 0.174	0.002
	Post-graduation	43 (56.6)	3.348 ± 0.635	

Practice settings	Hospital	46 (60.5)	3.152 ± 0.363	0.000
	Clinic	30 (39.5)	3.300 ± 0.651	
HCPs	Cardiologist	42(55.3)	3.381 ± 0.582	0.000
	Pharmacist	34 (44.7)	3.00 ± 0.246	
Experience	Less than 1 year	22 (28.9)	3.318 ± 0.567	0.000
	1-5 years	37 (48.7)	3.081 ± 0.363	
	6-10 years	17 (22.4)	3.352 ± 0.606	
**Total Knowledge score**		76 (100)	3.210 ± 0.498	

This study indicates that the fundamental knowledge of GDMT implementation between pharmacists and cardiologists is moderate. The accuracy percentages for the four GDMT knowledge items varied from 22.7% to 80.25% (Table 2). The average score of knowledge was 3.210 (SD: 0.498, range: 0–4), indicating an overall accurate rate of 59% (3.21/4*100) on this assessment. Knowledge scores exhibited substantial differences across pharmacists and cardiologists (P = 0.000), age categories (P = 0.000), educational level (P = 0.002), practice environments (P = 0.000), and levels of experience (P = 0.000) (Table 1). The understanding of current clinical recommendations for the care of IHD was highest at 80.25%, whereas awareness of recent updates to GDMT in IHD management over the past two years was lowest at 22.7%. No substantial difference was observed between genders for knowledge scores (Table 1). Correlation of knowledge regarding GDMT implementation between cardiologists and pharmacist are presented in Figure 1.

Table 2: Summary of questions for Knowledge, Attitudes and Practices towards GDMT. The correct answer is presented in percentage

**Questions**

**Knowledge of Guideline-directed Medical Treatment (GDMT)**
Q1: Do you familiar with the current clinical guidelines for the management of IHD (e.g., ACC/AHA guidelines)? (80.25%)
Q2: Do you familiar with key components (e.g. antiplatelet therapy, beta blockers, ACE inhibitors or ARBs, statins and lifestyle modifications) of Guideline-Directed Medical Therapy? 55.2%
Q3: Are you aware of recent updates to GDMT in IHD management in the last 2 years? 22.7%
Q4: Are you aware of the specific role of clinical pharmacists in implementing guideline-directed medical treatment? 56.2%
**Attitude**
A1: Do you think adhering to GDMT is IMPORTANT for improving patient outcomes in IHD? 44.6%
A2: is it feasible to implement GDMT in routine clinical practice for IHD patients? 91.5%
A3: Do you believe that pharmacist involvement in the care of IHD patients improves adherence to GDMT? 78.2%
A4: re you confident in the current multidisciplinary team approach to managing IHD, including cardiologists and pharmacists? 60.1%
What barriers, if any, do you perceive in implementing GDMT in your practice? (Select all that apply)<br>• Lack of patient adherence 25%<br>• Limited time in consultations 47.4%<br>• Complexity of guidelines 15.8%<br>• Insufficient support from other healthcare professionals 11.8%
**Practice**
P1: Do you believe that having a clinical pharmacist involved enhances patient education and medication adherence? 92.5%
P2: In your practice, do you often adjust a patient's medication regimen based on the GDMT guidelines for IHD? 74.01%
P3: Do you regularly involve pharmacists in decision-making for IHD patient treatment plans? 34.2%
P4: Do you frequently reassess a patient's GDMT based on changes in their clinical condition? 55.1%

P5: How frequently do you reassess a patient's GDMT based on changes in their clinical condition?

- Every 6 months (16.1%)
- Annually (22.4%)
- Only when symptoms worsen (45%)
- Never (16.5%)

P6: Which of the following methods do you use to assess patient adherence to GDMT?

- Direct questioning during follow-up (56.5%)
- Pill count (10.2%)
- Pharmacy refill data (22.7%)
- Medication adherence apps/tools (10.6%)

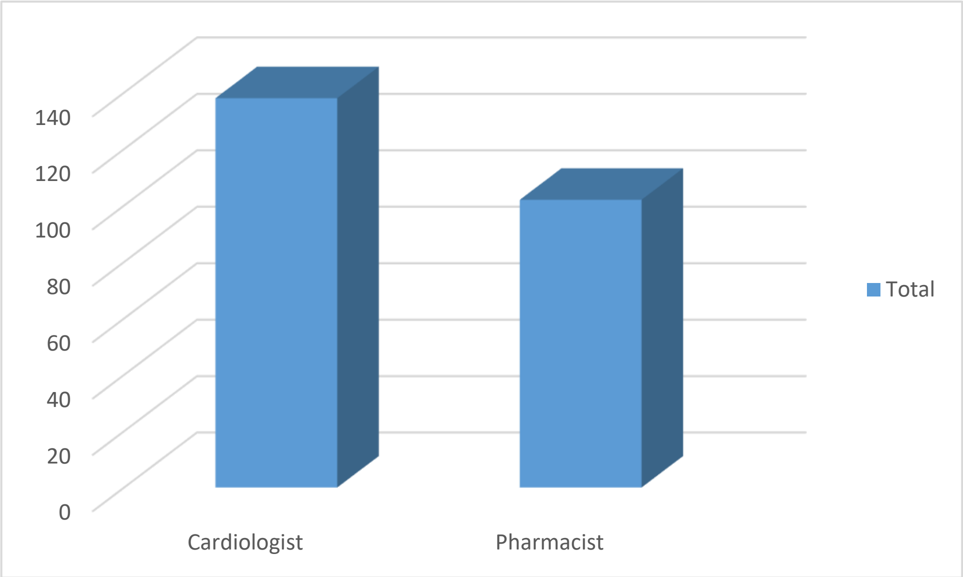

Figure 1. Correlation of knowledge regarding GDMT implementation between cardiologists and pharmacist

The majority of respondents agreed that implementing GDMT in ordinary clinical practice is possible, and that pharmacist's engagement enhances adherence to GDMT among IHD patients, with scores of 91.5% and 78.2%, respectively (Table 2). 40.1% of research participants expressed confidence in the existing multidisciplinary team strategy for managing IHD, which includes cardiologists and pharmacists. A restricted duration during consultations (47.4%) was regarded as a significant obstacle to the implementation of GDMT in reality.

Attitude scores exhibited a significant difference between graduate and post-graduate participants ($P < 0.05$) (Table 3). Correlation of attitude regarding GDMT implementation between cardiologists and pharmacist are presented in figure 2.

Table 3: The score of attitude towards GDMT by demographic variables

**Variables**	**Categories**	**N (%)**	**Attitude score (mean ± standard deviation)**	**P value**
Gender	Male	35 (46.1)	4.943 ± 0.726	0.056
	Female	41 (53.9)	3.21 ± 0.838	
Age in year	22-27	22 (28.9)	4.545 ± 1.010	0.101
	28-33	39 (51.3)	4.692 ± 0.799	
	34-39	15 (19.7)	4.333 ± 0.723	
Educational level	Graduation	33 (43.4)	4.818 ± 0.635	0.001
	Post-graduation	43 (56.6)	34.395 ± 0.954	
Practice settings	Hospital	46 (60.5)	4.673 ± 0.761	0.55
	Clinic	30 (39.5)	4.433 ± 0.971	
HCPs	Cardiologist	42(55.3)	4.619 ± 0.909	0.399
	Pharmacist	34 (44.7)	4.529 ± 0.787	
Experience	Less than 1 year	22 (28.9)	4.454 ± 0.911	0.086
	1-5 years	37 (48.7)	4.837 ± 0.764	
	6-10 years	17 (22.4)	4.176 ± 0.808	
**Total Attitude score**		76 (100)	4.578± 0.853	

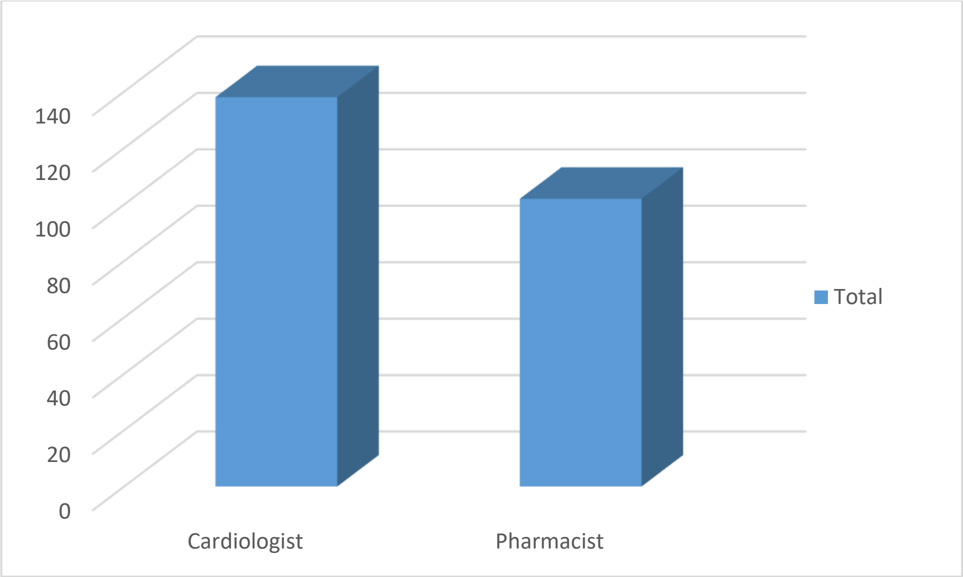

Figure 2. Correlation of attitude regarding GDMT implementation between cardiologists and pharmacist

The average practice score was 5.921 (SD: 1.05, range: 0 to 8). The highest practice score, achieved by participants over 50 years of age, was 74.01% (6/8*100). Practice scores shown considerable variation based on educational level and practice setting (P = 0.00), age groups (P = 0.01), education levels (P = 0.015), occupations (P 0.05), and knowledge score (P = 0.00) (Table 4). Forty-five percent of study participants reevaluated a patient's GDMT solely in response to changes in clinical condition, whereas 56.5% examined patient adherence to GDMT through direct inquiry during follow-up (Table 2). Correlation of practice regarding GDMT implementation between cardiologists and pharmacist are presented in figure 3.

Table 4: The score of practices towards GDMT by demographic variables

Variables	Categories	N (%)	Perception score (mean ± standard deviation)	P value
Gender	Male	35 (46.1)	5.800 ± 1.023	0.73
	Female	41 (53.9)	6.024 ± 1.083	
Age in year	22-27	22 (28.9)	5.090 ± 0.750	0.05

	28-33	39 (51.3)	6.512 ± 0.942	
	34-39	15 (19.7)	5.600 ± 0.736	
Educational level	Graduation	33 (43.4)	5.697 ± 1.310	0.00
	Post-graduation	43 (56.6)	36.093 ± 0.781	
Practice settings	Hospital	46 (60.5)	5.826 ± 1.216	0.00
	Clinic	30 (39.5)	6.066 ± 0.739	
HCPs	Cardiologist	42(55.3)	6.071 ± 0.894	0.05
	Pharmacist	34 (44.7)	5.735 ± 1.213	
Experience	Less than 1 year	22 (28.9)	5.727 ± 1.202	0.00
	1-5 years	37 (48.7)	5.882 ± 0.696	
	6-10 years	17 (22.4)	4.235 ± 0.970	
Total Perceptions score		76 (100)	5.921 ± 1.055	

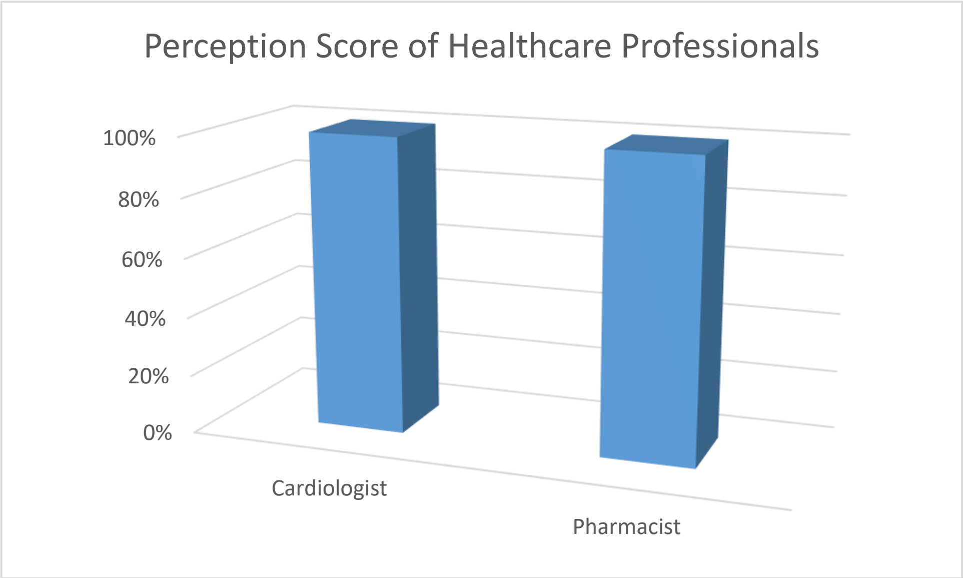

Figure 3. Correlation of practice regarding GDMT implementation between cardiologists and pharmacist

However, the outcomes variables of the multiple linear regression analysis exhibiting low scores on KAP indicators indicate a substantial difference in knowledge of GDMT between cardiologists and pharmacists (β: 0.383, P = 0.001), which was significantly correlated with

moderate knowledge scores, while pharmacists demonstrated a lower attitude score compared to cardiologists (β: 0.094, P = 0.001). Moreover, no substantial difference was observed in the practice of implementation of GDMT between pharmacists and cardiologists (β: 0.051, P > 0.005). The power value (0.949) also indicates a significant relationship between HCPs. (Table 5).

Table 5. Association of sociodemographic variables with GDMT of knowledge, attitude and practice by multiple linear regression

**Variables**	**Coefficient (β)**	**Standard error**	**t**	**P value**	**Power analysis (1- β)**
**Knowledge**					
Age in year	o.144	0.164	1.22	0.224	0.856
Educational status	0.251	0.112	2.164	0.034	0.749
HCPs	0.383	0.114	3.385	0.001	0.617
Experience	0.035	0.176	0.288	0.774	0.965
Practice settings	0.098	0.119	0.814	0.419	0.902
**Attitude**					
Age in year	0.093	0.095	0.793	0.430	0.907
Education	0.251	0.065	2.253	0.027	0.749
HCPs	0.094	0.066	0.835	0.001	0.906
Experience	0.113	0.102	0.929	0.356	0.887
Practice settings	0.149	0.069	1.251	0.215	0.851
**Practice**					

Age in year	0.229	0.078	1.922	0.059	0.771
Education	0.180	0.054	1.588	0.117	0.82
HCPs	0.051	0.054	0.443	0.659	0.949
Experience	0.094	0.084	0.761	0.449	1.094
Practice settings	0.119	0.057	0.977	0.322	0.8

Discussion

Key findings

This study highlights important insights into the knowledge and practices of HCPs, including cardiologists and pharmacists, regarding the implementation of GDMT for IHD. This study also found that HCPs had moderate knowledge of the implementation of GDMT in healthcare settings. However, there was a substantial knowledge difference in the implementation of GDMT between cardiologists and pharmacists. The understanding of current clinical recommendations for the care of IHD was the highest, whereas awareness of recent updates to GDMT in IHD management over the past two years was the lowest among study participants. The majority of the study participants expressed confidence in the existing multidisciplinary team strategy for managing IHD, which included cardiologists and pharmacists. In reality, a restricted duration during consultations was regarded as a significant obstacle to the implementation of GDMT.

Strength and weakness of study

This study represents the first investigation of the knowledge, attitudes, and practices of HCPs regarding the implementation of GDMT in patients with IHD. Through an examination of the perspectives and behaviors of these key healthcare professionals, this research sought to identify knowledge gaps, potential misconceptions, and practical challenges that may

hinder the optimal utilization of GDMT. Additionally, the study aimed to provide a healthcare team with insights into the implementation and updating of GDMT. Moreover, this investigation identified several barriers that must be addressed to ensure high-quality care. This study had certain limitations. The small sample size is a major limitation of this study because of the single study center, and most healthcare professionals refused to participate in the survey due to their busy schedules. The absence of an independent interviewer may also be considered a limitation of this study. The interviewer was acquainted with the majority of participants prior to this study, and all participants were aware that they were pharmacists with the objective of developing medicine review services. This may have influenced participants' responses to certain questions.

Interpretation

The management of ischemic heart disease (IHD) is a complex process that requires a multidisciplinary approach to optimize patient outcomes [6]. Clinical pharmacists play a crucial role alongside cardiologists as healthcare teams in implementing GDMT for individuals with IHD. The partnership between clinical pharmacists and cardiologists is effective in the implementation of GDMT in patients with IHD. To the best of our knowledge, there is a lack of studies examining healthcare professionals' knowledge, attitudes, and practices (KAP) of healthcare professionals regarding the implementation of guideline-directed medical therapy (GDMT). Our study revealed that approximately 60% of cardiologists and pharmacists demonstrated modest knowledge of GDMT implementation based on their responses to knowledge-related questions. The study participants exhibited moderate knowledge, but poor practices concerning GDMT implementation for IHD. However, our findings are consistent with a previous cross-sectional study that indicated that Beijing physicians had inadequate knowledge and practices in relation to GDMT for HFrEF [13].

Moreover, our findings highlight a significant disparity in the knowledge of cardiologists and pharmacists. The current clinical recommendations for IHD care were high, and awareness of recent GDMT updates in IHD management over the past two years was the lowest among HCPs. In contrast, cardiologists exhibited a strong awareness of and a favorable attitude towards the understanding of ESC guidelines for congestive heart failure (CHF) recommendations and believed that one of the most important ways to enhance implementation is to provide recommendations in a clear and understandable manner [20]. However, the HCPs in our study perceived that limited time in consultations is a major barrier to the implementation of GDMT in healthcare settings. This finding is aligned with a previous survey, in which more than half (53.4%) of cardiologists perceived consultation time as a significant barrier to guideline adherence [21]. Moreover, these findings are also consistent with another study in which 26% of doctors believed that time constraints during consultations were a system-based barrier to GDMT implementation [22].

Nevertheless, research has also revealed that the inclusion of dedicated pharmacists and nursing teams in inpatient heart failure (HF) units correlates with slightly higher rates of GDMT implementation [23]. These findings are consistent with our results that most HCPs agreed that GDMT could be effectively applied in routine clinical settings in the presence of pharmacists and cardiologists. In addition, the majority of participants in our study also believed that pharmacist involvement in the healthcare team improves GDMT adherence in IHD patients. These findings are consistent with previous findings that the inclusion of a clinical pharmacist and nurse specialist in a multidisciplinary heart failure program had a beneficial impact on patient care. [24]. Moreover, the current results showed that some pharmacists (34.2%) were regularly involved in decision-making regarding IHD patient treatment plans. Our findings also show that almost half (55.1%) of the HCPs frequently reassessed a patient's GDMT based on changes in their clinical condition. The majority of HCPs (92.5%) believed that having a

clinical pharmacist involved in the healthcare team enhanced patient education and medication adherence. These results are consistent with those of a previous study reporting that the involvement of pharmacists in a multidisciplinary team could optimise patients' HF and non-HF medications by a comprehensive review of all prescribed drugs. These factors are extremely important [25]. However, our findings support the development of education and training programs for pharmacists to improve the management of IHD by a multidisciplinary team. The study underscores the significance of targeted interventions to address KAP gaps, advocating for enhanced educational initiatives, and formalized collaboration frameworks between cardiologists and pharmacists.

Future research

Improved adherence to GDMT through augmented interdisciplinary cooperation could play a crucial role in optimising the care of patients with IHD. Subsequent research should investigate the long-term impact of such interventions on patient outcomes and evaluate the efficacy of structured training programs in mitigating the KAP gaps in clinical practice. Subsequent investigations will elucidate strategies to enhance multidisciplinary collaboration and improve adherence to evidence-based treatment guidelines, ultimately contributing to improved clinical outcomes in patients with IHD.

Declaration

Acknowledgement

We are highly thankful to the Department of Pharmacy of Quaid-i-Azam University, Islamabad and Punjab Institute of Cardiology, Lahore for the support to conduct this study.

Author contribution

All authors (UK, SSS, AK, AI, SMA, and YF) designed and conceptualized the study. AI reviewed and validated the study questionnaire. UK and SMA collected the data. UK and SSS made substantial contributions to the data analysis. U.K. drafted the manuscript, and AK and AI made significant revisions. All authors have read and approved the final manuscript. A.K. is the study's supervisor.

Funding

No funding was received for this study.

Data availability

All data generated or analyzed during this study are included in this published article

Ethical approval and consent to participate

This study was approved by the Bioethics Committee of the Faculty of Biological Sciences at Quaid-i-Azam University, Islamabad (ref. #: BEC-FBS-QAU2021-270) and the ethical review committee of the Punjab Institute of Cardiology (PIC), Hospital in Lahore (ref. #: RTPGME-Research-204) in compliance with the Declaration of Helsinki and the Australian New Zealand registry. PIC granted a waiver of consent for the collection of administrative data for each randomized participants. Written informed consent was also obtained prior to enrollment in the study.

Consent to participate

Written and oral informed consent was obtained from all individual participants included in the study.

Consent for publication

Not applicable

Competing interest

The authors declare that they have no conflict of interest.

References

1. Kyavar, M. and M. J. Alemzadeh-Ansari (2022). Stable ischemic heart disease. Practical cardiology, Elsevier**:** 429-453

2. Benjamin, E. J., et al. (2019). "Heart disease and stroke statistics—2019 update: a report from the American Heart Association." Circulation **139**(10): e56-e528.

3. Roth, G. A., et al. (2020). "Global burden of cardiovascular diseases and risk factors, 1990–2019: update from the GBD 2019 study." Journal of the American College of Cardiology **76**(25): 2982-3021
4. Dababneh, E. and Goldstein, S., 2018. Chronic ischemic heart disease selection of treatment modality.
5. Lewis, J.M. and Davis, L.L., 2013. Management of stable ischemic heart disease. *The Journal for Nurse Practitioners*, *9*(10), pp.661-668.
6. Sarraju, A., Maron, D.J. and Rodriguez, F., 2019. Under-Reporting and Under-Representation of Racial/Ethnic Minorities in Major Atrial Fibrillation Clinical Trials. *Circulation*, *140*(Suppl_1), pp.A15956-A15956.
7. Alibasic, E., Ljuca, F., Batic-Mujanovic, O., Ramic, E., Bajraktarevic, A. and Alibasic, E.E., Therapeutic Modalities in Patients with Ischemic Heart Disease.
8. Agbor-Etang, B.B. and Setaro, J.F., 2015. Management of hypertension in patients with ischemic heart disease. *Current cardiology reports*, *17*, pp.1-7.

9. Crosier, R., Austin, P.C., Ko, D.T., et al., 2021. Intensity of guideline-directed medical therapy for coronary heart disease and ischemic heart failure outcomes. *The American Journal of Medicine*, *134*(5), pp.672-681.
10. Mohiuddin, A.K., 2020. The excellence of pharmacy practice. *INNOVATIONS in pharmacy*, *11*(1).
11. Fihn SD, Gardin JM, Abrams J, et al. 2012 ACCF/AHA/ACP/AATS/PCNA/SCAI/STS Guideline for the Diagnosis and Management of Patients with Stable Ischemic Heart Disease. *Circulation.* 2012;126(25):e354-e471.
12. Shahid, I., Khan, M.S., Fonarow, G.C., Butler, et al., 2024. Bridging gaps and optimizing implementation of guideline-directed medical therapy for heart failure. *Progress in Cardiovascular Diseases*.
13. Cox, Z.L., Nandkeolyar, S., Johnson, A.J., et al., 2022. In-hospital initiation and up-titration of guideline-directed medical therapies for heart failure with reduced ejection fraction. *Cardiac Failure Review*, *8*.
14. Devoldere, J., Droogmans, S., Heggermont, et al., 2024. Implementation of guideline-directed medical therapy for heart failure patients with reduced ejection fraction in Belgium: a Delphi panel approach. *Acta Cardiologica*, pp.1-12.
15. Polit, D.F. and Beck, C.T., 2006. The content validity index: are you sure you know what's being reported? Critique and recommendations. *Research in nursing & health*, *29*(5), pp.489-497.
16. Kaliyaperumal, K.I.E.C., 2004. Guideline for conducting a knowledge, attitude and practice (KAP) study. *AECS illumination*, *4*(1), p.7.

17. Olum, R., Chekwech, G., Wekha, G., et al., 2020. Coronavirus disease-2019: knowledge, attitude, and practices of health care workers at Makerere University Teaching Hospitals, Uganda. *Frontiers in public health*, *8*, p.181.
18. Seid, M.A. and Hussen, M.S., 2018. Knowledge and attitude towards antimicrobial resistance among final year undergraduate paramedical students at University of Gondar, Ethiopia. *BMC infectious diseases*, *18*, pp.1-8.
19. Koo, T.K. and Li, M.Y., 2016. A guideline of selecting and reporting intraclass correlation coefficients for reliability research. *Journal of chiropractic medicine*, *15*(2), pp.155-163.
20. Erhardt, L., Komajda, M., Hobbs, F.R. et al., 2008. Cardiologists' awareness and perceptions of guidelines for chronic heart failure. The ADDress your Heart survey. *European journal of heart failure*, *10*(10), pp.1020-1025.
21. McKee, G., Kerins, M., Hamilton, G., et al., 2017. Barriers to ESC guideline implementation: results of a survey from the European Council on Cardiovascular Nursing and Allied Professions (CCNAP). *European journal of cardiovascular nursing*, *16*(8), pp.678-686.
22. Vasudevan, S., Thayaparan, A., Teng, L.E., Lamoza, N., et al., 2024. Physician perceptions of medication prescribing in heart failure: a scoping.
23. Zheng, J., Mednick, T., Heidenreich, P.A. et al., 2023. Pharmacist-and nurse-led medical optimization in heart failure: a systematic review and meta-analysis. *Journal of Cardiac Failure*, *29*(7), pp.1000-1013.
24. AlHabeeb, W., Alayoubi, F., Hayajneh, A., et al., 2024. A strategy to improve adherence to guideline-directed medical therapy (GDMT) and the role of the multidisciplinary team in a heart-failure programme. *Cardiovascular Journal of Africa*, *35*(1), pp.12-15.

25. Campbell, G., Doherty, C., D'Silva, A., Carr-White, G., et al., 2024. Implementation and evaluation of specialist heart failure pharmacist prescribing clinics. *International Journal of Clinical Pharmacy*, pp.1-7.

Click here to access/download
Supporting Information
Questionnaire.pdf

Click here to access/download
Supporting Information
STROBE_checklist_cross-sectional.docx
