# Supplementary material for: Implementation of guideline-directed medical treatment for ischemic heart disease management: A knowledge, attitude and practice based cross-sectional survey
Source: PLoS One. 2026 Feb 4;21(2):e0338634. doi: 10.1371/journal.pone.0338634 (PMC12872007; doi:10.1371/journal.pone.0338634)
Supplement: Attachment — Submitted filename: Response to Reviewers-PLOS ONE.docx [file pone.0338634.s008.docx]

**Response to Editor and Reviewers' Comments (PLOS ONE- Manuscript ID: PONE-D-25-18404)**

Dear Editor, we would like to express our gratitude for the swift handling of our manuscript and facilitating its peer review. We also thankful for giving us the opportunity to submit a revised draft of our manuscript. We are very glad to receive such extensive comments from Reviewers. The comments from Reviewers demonstrated a high level of expertise and acknowledgment of the subject. We appreciate the time and effort that you and the reviewers have dedicated to providing your valuable feedback on our manuscript.

Kindly find enclosed herewith, a revised version of our manuscript entitled “**Implementation of Guideline-Directed Medical Treatment for Ischemic Heart Disease Management: A Knowledge, Attitude and Practice Based Cross-Sectional Survey**” for re-evaluation and publication in	***PLOS ONE***. We have addressed all of the comments and included some major revisions and edits to our work. Sometimes, we had slightly different opinions than Reviewers, but we did our best to respond to the comments and still provide some changes in the manuscript for better justification and understanding of the approach undertaken.

**Response to Reviewer 1**

We are thankful to the reviewer for evaluation of our manuscript. All the suggestions have been incorporated in revised manuscript. Reviewers’ comments were very helpful to incorporate the updated information. All the points raised by the learned reviewer have been addressed. We do hope that these changes will make points clear and will improve the overall presentation of our manuscript.

A point-by-point response to the reviewers’ comments and concerns:

**Query #1:**

This study addresses an important area regarding the implementation of Guideline-Directed Medical Therapy (GDMT) implementation for ischemic heart disease (IHD) within resource-limited settings. The finding that "this is the first investigation of the knowledge, attitudes, and practices of HCPs regarding the implementation of GDMT in patients with IHD" is a strong point. However, the manuscript needs to be strengthened in terms of methodological rigor, statistical reporting, and clarity of presentation. Limitations include a single-center design (Punjab Institute of Cardiology) and small convenience sample (n=76), restrict generalizability and risk selection bias. Pharmacist (n=34) versus cardiologist (n=42) comparisons may lack statistical power, while inconsistent practice metrics (e.g., citing highest scores for participants over 50 years despite Table 4 excluding this age group) and absent correlation figures weaken the evidence for healthcare professional (HCP) differences. Broader claims about collaborative care necessitate multi-center validation.

**Response:**

We sincerely thank the reviewer for the constructive feedback and for recognizing the significance of this study in addressing GDMT implementation within a resource-limited setting. We appreciate the comments regarding the methodological and statistical aspects and have made several revisions to enhance the clarity, rigor, and transparency of the manuscript.

**Study Design and Generalizability:**

We fully acknowledge the limitations associated with the single-center design and the use of convenience sampling. These constraints are now explicitly discussed in the Limitations section (Page # 32, Line # 532-534). We have also cited the relevant literatures for small sample size in sample size of method section to substantiate the implications of non-probability sampling on external validity (Page # 10, Line # 178-190)

We have revised the results tables and text to include full reporting of statistical tests, including:

Pearson correlation coefficients (r), degrees of freedom, and p-values, ANOVA F-values, degrees of freedom, and post hoc test results (Tukey’s HSD) in the revised manuscript. The results are now reported with greater caution, without overstating between-group differences. Where applicable, we have reported effect sizes alongside p-values to contextualize the magnitude of observed associations in result section (Page # 13-28, Line # 263-431).

Moreover, clarification of how each test was applied to specific variables (e.g., independent samples t-test for binary variables, ANOVA for variables with ≥3 groups, MLR for multivariable associations) were explained in method section (Page # 12-13- , Line # 238-254)

**Clarification of Age Group Reporting:**

We appreciate the reviewer’s observation regarding inconsistencies in reported age-related findings. Upon review, we found that the reference to participants “over 50 years” was mistakenly retained from an earlier draft. This has now been corrected, and the text is aligned with the actual groupings used in all tables. All references to age-specific analyses have been cross-checked for consistency.

**Interpretation and Claims Regarding Collaborative Care:**

We agree that broader claims about pharmacist–cardiologist collaboration must be made with caution. Accordingly, we have revised the discussion to frame these as emerging observations consistent with international trends, but not definitive conclusions (Page # 28-31, Line # 432-508). We emphasize the need for multi-center, interventional research to evaluate structured collaborative care models in the context of IHD in recommendation for future research section (Page # 33, Line # 547-549).

We believe that these revisions significantly improve the scientific and interpretive rigor of the manuscript, and we are grateful to the reviewer for highlighting these important areas.

**Query # 2:**

Statistical methods (t-tests, ANOVA, regression) are generally appropriate for the cross-sectional design, and Bonferroni correction (α=0.0125) is correctly applied. However, critical anomalies undermine rigor: Table 3 reports implausible attitude scores (e.g., 34.395 ± 0.954 for post-graduates), suggesting data errors. Power analysis results are inconsistently interpreted (e.g., 0.949 for practice vs. 0.617 for knowledge) without justification for variable thresholds, and β-values (e.g., β=0.383 for knowledge disparity) lack effect-size context (e.g., Cohen’s), obscuring clinical significance. Additionally, while the manuscript claims all data are included, only supplementary materials (questionnaires, STROBE checklist) are accessible, and raw datasets are unavailable. Authors should rectify statistical tables and share de-identified data via repositories.

**Response:**

We are grateful for the reviewer’s detailed assessment of our statistical methods and reporting. We have carefully addressed each of the concerns raised and have made substantive revisions to the manuscript to enhance transparency, validity, and reproducibility.

**Correction of Implausible Values in Table 3:**

We appreciate the reviewer’s identification of implausibly high attitude scores (e.g., 34.395 ± 0.954 for post-graduates). Upon review, we discovered a data entry error in the attitude score computation due to the incorrect aggregation of Likert scale items. The issue has now been rectified, and comparison of GDMT-Related Attitude Scores across Demographics has been revised accordingly and named as Table 5 (Page # 20, Line # 344-346) and Table 6 Page # 21, Line # 358-360) in the manuscript.

**Clarification of Power Analysis Interpretation:**

The concern regarding the inconsistent interpretation of power analysis (e.g., 0.949 for practice vs. 0.617 for knowledge) is valid. We have, now, revised the results section to include interpretation of effect sizes for β-values derived from multiple linear regression. Specifically, Cohen’s f² values were calculated to contextualize the strength of associations (e.g., small: 0.02, medium: 0.15, large: 0.35). This contextualization now appears in the results and is discussed with respect to clinical relevance. (Page # 24-28, Line # 395-431)

**Data Availability Statement and Repository Submission:**

In response to the reviewer’s point on data accessibility statement, the manuscript and its Supporting Information files contain the minimal data set used to reach the conclusions presented in the study. However, the revised manuscript includes an updated **Data Availability Statement, “**All relevant data are within the paper and its Supporting Information files”**.** (Page # 34, Line # 585-586).

We hope this satisfies the journal’s requirements for data transparency and reproducibility.

We thank the reviewer once again for these critical recommendations, which have significantly strengthened the methodological rigor and transparency of the revised manuscript.

**Query # 3:**

The manuscript is generally clear but contains grammatical errors (e.g., "re you confident" corrected to "Are you confident"; inconsistent "perception" vs. "practice" scoring) and ambiguous phrasing (e.g., contradictory β-values describing attitude differences). Terminology like "bad KAP" should be standardized to "poor KAP." Ethically, approval and consent procedures are documented, though interviewer-participant familiarity risks social desirability bias acknowledged by authors. As the first Pakistani KAP study on GDMT, it valuably highlights barriers (e.g, time constraints) and aligns with global calls for multidisciplinary collaboration. However, small sample size limits subgroup analyses, and qualitative insights into poor GDMT adoption are absent. Revisions should clarify statistical anomalies, propose barrier-mitigation strategies (e.g., telehealth), and contextualize findings via LMIC comparisons.

**Response:**

We thank the reviewer for their constructive feedback and recognition of the study’s contribution as the first KAP survey on GDMT implementation in Pakistan. We have addressed each of the identified issues as follows:

**Grammatical and Terminological Corrections:**

We have reviewed the manuscript thoroughly and corrected typographical and grammatical errors. For example, "re you confident" has been corrected to "Are you confident." Inconsistencies in terminology have also been addressed—"bad KAP" has been revised to "poor KAP" throughout the manuscript to reflect standard academic language. Additionally, we have ensured consistent reference to "practice" scores instead of "perception" where appropriate.

**Clarification of Ambiguous Statistical Phrasing:**

We have revised the results and discussion sections to address ambiguous interpretations of β-values. Contradictory statements regarding the direction and magnitude of associations have been clarified. Each statistically significant predictor is now interpreted alongside its directionality and corresponding effect size (Cohen’s f²), with inconsistent or non-significant findings explicitly described to avoid misinterpretation. To enrich interpretation, we integrated comparisons with GDMT implementation literature from LMIC settings in Discussion section (Page # 13-32, Line # 263-524)

**Ethical Considerations and Interviewer Bias:**

We appreciate the reviewer’s point regarding social desirability bias due to interviewer-participant familiarity. This concern has now been explicitly discussed under study limitations, including the potential impact on self-reported responses. While ethical approval and informed consent were obtained, we acknowledge that the lack of an independent interviewer may have influenced participants' disclosure and this has been framed accordingly. (Page # 32, Line # 541-545)

**Sample Size and Subgroup Analysis Limitations:**

Thank you for this insightful comment. We have addressed the limited sample size in the revised method section, acknowledging its impact on subgroup analysis and interpretive confidence in the discussion section as already mentioned above.

Furthermore, we discussed strategies such as pharmacist-led telehealth models and structured education programs as feasible interventions to improve adherence in resource-limited environments in implication for practice section (Page # 31-32, Line # 519-522)

Lastly, we recognized the absence of qualitative data as a limitation and have proposed mixed-methods research to address this in recommendation for future studies. (Page # 33, Line # 562-564).

We believe these revisions significantly improve the manuscript’s clarity, methodological transparency, and policy relevance. We are grateful for the reviewer’s helpful comments and guidance.

**Response to Reviewer 2**

**Query # 1:**

 The manuscript requires major revisions, particularly, the statistical analysis and interpretation. The research question was not stated explicitly nor were subquestions identified. A hypothesis was absent although the research design and methodology were provided. The authors did not elaborate on the rationale for the statistical analyses chosen for the study or specify the groups that were being compared and it is unclear which component of the study the correlation, ANOVA, and multiple linear regression were applied. The interpretation is fairly weak. The authors simply restate their results and state, "the results are consistent" but do not expound on what their interpretation of these consistencies are, the implications for the study, or the implications for the field at large. The discussion should be robust in which they are expounding on the significance and implications. Furthermore, it is an overestimation to say what is consistent with previous findings while only citing one finding per result in the present study. If feedback is implemented, rigor, transparency, reproducibility, and application can be enhanced.

**Response:**

**Clarification of Research Question, Sub-questions, and Hypothesis**

We agree that the original manuscript did not clearly state the central research question and supporting sub-questions. We have now revised the Introduction to explicitly include the following:

**Research Question**: What are the levels of knowledge, attitudes, and practices regarding GDMT among cardiologists and clinical pharmacists, and what demographic or institutional factors influence these behaviors in a Pakistani tertiary care setting? (Page # 7, Line # 129-130).

**Sub-questions**: (Page # 7-8, Line # 131-135).

- Are there significant differences in KAP scores between professional roles (cardiologists vs. pharmacists)?
- Which sociodemographic factors (e.g., age, gender, qualification, experience) are associated with KAP domains?
- To what extent do these factors predict knowledge, attitude, and practice related to GDMT?

A corresponding set of **hypotheses** has also been added: (Page # 8, Line # 136-140).

- **H₁**: There is a significant difference in KAP scores between cardiologists and pharmacists.
- **H₂**: Sociodemographic characteristics significantly predict KAP scores.
- **H₀**: There are no significant differences or associations in KAP domains based on professional role or demographic factors.

**2. Justification and Clarification of Statistical Methods**

We appreciate the need for clearer justification of the statistical tests used. We have now elaborated in the Methods section with the following revisions:

- **Independent samples t-tests** were used to compare mean KAP scores between binary groups (e.g., gender, HCPs).
- **One-way ANOVA** was applied to variables with more than two categories (e.g., qualification levels), followed by **Tukey’s HSD** for post hoc comparisons where significance was detected.
- **Pearson correlation analysis** was used to examine associations between continuous variables and KAP domains.
- **Multiple linear regression (MLR)** was used to evaluate the independent and combined effects of sociodemographic predictors on each KAP outcome (knowledge, attitude, and practice), adjusting for confounding.

All analyses have now been explicitly aligned with specific research objectives in the Statistical Analysis subsection. (Page # 12-13, Line # 238-254)

**3. Improved Interpretation of Findings**

We acknowledge that the initial discussion was overly descriptive. We have significantly revised the Discussion section to move beyond restating results. We also agree that citing a single study per finding does not adequately demonstrate alignment with the broader literature. We have expanded the references and now situate our findings within regional and international contexts, drawing on relevant studies from low- and middle-income countries (LMICs) and recent systematic reviews.

We have updated the manuscript to improve transparency and reproducibility, including:

- Clearly stated research questions and hypotheses (Page # 7-8, Line # 129-140)
- Full reporting of statistical assumptions and effect sizes (e.g., Cohen’s d and f²) and revised and labeled regression models to distinguish between full and reduced models in the result section (Page # 24-28, Line # 395-431)
- We have also incorporated implications for practice in the Discussion section. (Page # 31-32, Line # 508-524)

**Interpretation and discussion**: We have extensively revised the **Discussion** to go beyond restating the results. We now interpret the findings in the context of existing literature and practice implications. We have also:

- Elaborated on the meaning of statistically significant and non-significant results.
- Provided **at least 2–3 references per key finding** to avoid overgeneralization.
- Highlighted the implications for clinical practice, interdisciplinary collaboration, and policy in low- and middle-income settings.
- Discussed limitations and proposed directions for future research.

**Consistency with previous findings**: We have revised language throughout the manuscript to avoid overstatements and ensure that comparisons with prior literature are adequately supported by multiple citations. In cases where findings align with prior work, we now provide **specific references** and context (Page # 28-32, Line # 432-524).

We believe these revisions strengthen the manuscript's rigor, interpretation, and contribution to implementation science in IHD care. We thank the reviewer once again for the insightful recommendations that guided these improvements.
