# Supplementary material for: Implementation of guideline-directed medical treatment for ischemic heart disease management: A knowledge, attitude and practice based cross-sectional survey
Source: PLoS One. 2026 Feb 4;21(2):e0338634. doi: 10.1371/journal.pone.0338634 (PMC12872007; doi:10.1371/journal.pone.0338634)
Supplement: Attachment — Submitted filename: Response_to_Reviewers-PLOS_ONE_auresp_2.docx [file pone.0338634.s009.docx]

**Response to Editor and Reviewers' Comments (PLOS ONE- Manuscript ID: [PONE-D-25-18404R1] - [EMID:3474814d2fe6b002]**

Dear Editor,

We sincerely thank you for the thoughtful evaluation of our manuscript and the constructive feedback provided. We appreciate the recognition of our study’s merit and are grateful for the opportunity to revise and resubmit.

We would also like to sincerely thank all reviewers for their time, constructive feedback, and thoughtful suggestions, which have greatly improved the clarity, methodological rigor, and overall quality of our manuscript. We carefully addressed each point raised, including refining the research questions and hypotheses, ensuring consistency between the statistical analysis and results, and expanding both the practical implications and recommendations. These revisions have substantially strengthened the manuscript and enhanced its contribution to the understanding of GDMT implementation in resource-limited settings. We are grateful for the opportunity to revise our work and trust that the current version meets the standards for acceptance.

Kindly find enclosed herewith, a revised version of our manuscript entitled “**Implementation of Guideline-Directed Medical Treatment for Ischemic Heart Disease Management: A Knowledge, Attitude and Practice Based Cross-Sectional Survey**” for re-evaluation and publication in	***PLOS ONE***. We have addressed all of the comments and included some minor revisions and edits to our work.

**Response to Reviewer 2**

We sincerely thank the reviewer for acknowledging the substantial revisions made in response to the earlier recommendations. We are also thankful to the reviewer for carefully noting the need to align our research questions, sub-questions, and hypotheses with the statistical analyses and results.

A point-by-point response to the reviewers’ comments and concerns:

**Query # 1**

The final recommendation is with regards to research question, sub-question, and hypotheses. Hypotheses are required for all research questions with the exception of those that are descriptive in nature. The researchers provided two additional subquestions; The first subquestion seeks to explore the association between sociodemographic factors and KAP domains which implies correlational (hypotheses required) and how sociodemographic factors predict KAP related to GDMT which implies regression (hypotheses required). Hypotheses were only provided for the first subquestion. Ensure that the description of these questions is consistent with the statistical analysis and description of results. This manuscript is acceptable once these changes are made.

**Response**

We have revised the “Research Questions and Hypotheses” section to ensure clarity and consistency across the manuscript (Page #8, Line #138-151). The final set of hypotheses is now as follows:

- **Sub-question 1 (Comparative):**

H₀₁: There are no significant differences in KAP scores between cardiologists and pharmacists.

H₁₁: There are significant differences in KAP scores between cardiologists and pharmacists.

- **Sub-question 2 (Associational):**

H₀₂: Sociodemographic factors are not significantly associated with KAP domains.

H₁₂: Sociodemographic factors are significantly associated with KAP domains.

- **Sub-question 3 (Predictive/Regression):**

H₀₃: Sociodemographic factors do not significantly predict knowledge, attitudes, or practices related to GDMT.

H₁₃: Sociodemographic factors significantly predict knowledge, attitudes, or practices related to GDMT.

These hypotheses are consistently reflected in the **Statistical Analysis** section (Page # 13-14, Line # 250-275), where each sub-question is mapped to the corresponding tests (e.g., t-tests/ANOVA for Sub-question 1, correlations/chi-square for Sub-question 2, and regression models for Sub-question 3). In addition, the **Results section** (Page #17-28, Line # 313- 479) has been revised to explicitly state whether the null or alternative hypotheses were retained or rejected for each analysis.

**I believe that these revisions ensure full consistency between the research questions, hypotheses, analyses, and results, thereby addressing the reviewer’s concerns and strengthening the methodological rigor of the manuscript.**

**Reviewer # 3:**

We sincerely thank the reviewer for recognizing the relevance and timeliness of our work. Our aim was to highlight the challenges and opportunities surrounding GDMT implementation in a resource-limited setting, and we appreciate the acknowledgment that this study contributes to both local and global discussions on improving cardiovascular care in LMICs.

**Reviewer’s Suggestion:**

The study provides valuable insights into GDMT implementation in a resource-limited setting; however, the single-center design in Pakistan and relatively small convenience sample (n=76) limit the generalizability of the findings. A larger, multi-center study would strengthen external validity (for future works)

**Response:**

We thank the reviewer for this thoughtful suggestion. We acknowledge that the single-center design and relatively small convenience sample limit the generalizability of the findings. This limitation has already been noted in the **Strengths and Limitations** section of the manuscript **(Page # 35, Line # 606-608)**. However, we expanded this line in limitation **(Page # 35, Line # 608-609).** In line with the reviewer’s advice, we have also further expanded this point in the **Future Work section (Page # 36-37, Line # 642-644)**, where we emphasize the need for larger, multi-center studies across diverse healthcare settings to enhance external validity and improve the broader applicability of the results.

**Reviewer’s Recommendation:**

1. Further refinement and proofreading would enhance clarity and ensure accurate communication of results.
2. Expand the practical implication (in the discussion) or add a recommendation section.

**Response:**

1. undergone further refinement and careful proofreading to improve clarity, precision, and readability.
2. To address the reviewer’s suggestion, we have also expanded the Discussion by adding a brief ***Recommendations subsection*** to highlight actionable strategies, including targeted professional training, institutional support for guideline dissemination, and system-level initiatives to promote GDMT uptake in resource-limited contexts (Page # 34, Line # 576-598). These changes strengthen the translational value of our work and ensure clearer communication of its relevance to practice and policy.
