# Supplementary material for: Implementation of guideline-directed medical treatment for ischemic heart disease management: A knowledge, attitude and practice based cross-sectional survey
Source: PLoS One. 2026 Feb 4;21(2):e0338634. doi: 10.1371/journal.pone.0338634 (PMC12872007; doi:10.1371/journal.pone.0338634)
Supplement: Attachment — Submitted filename: Response to Reviewers-PLOS ONE -.docx [file pone.0338634.s010.docx]

**Response to Editor and Reviewers' Comments (PLOS ONE- Manuscript ID: [PONE-D-25-18404R2] - [EMID:58b69c9c58f6d2ae]**

Dear Editor,

We sincerely thank you and the reviewers for your valuable time and constructive feedback on our manuscript entitled *“****Guideline-Directed Medical Treatment for Ischemic Heart Disease Management: A Knowledge, Attitude and Practice Based Cross-Sectional Survey****”* (Manuscript ID: [PONE-D-25-18404R2]-[EMID:58b69c9c58f6d2ae]). We are encouraged by your assessment that the manuscript has merit, and we appreciate the opportunity to revise and resubmit it for further consideration in *PLOS ONE*.

We have carefully addressed all comments raised by the reviewers and editorial team. Each point has been considered with great care, and corresponding revisions have been incorporated throughout the manuscript to enhance its clarity, methodological rigor, and overall scientific quality.

A detailed **point-by-point response document** has been prepared, outlining how each comment was addressed. All revisions in the main manuscript are highlighted using yellow highlights for ease of review.

We believe that the revised version now meets the publication criteria of *PLOS ONE* and hope that it will be found suitable for publication.

Kindly find enclosed herewith, a revised version of our manuscript entitled “**Implementation of Guideline-Directed Medical Treatment for Ischemic Heart Disease Management: A Knowledge, Attitude and Practice Based Cross-Sectional Survey**” for re-evaluation and publication in	***PLOS ONE***. We have addressed all of the comments and included some minor revisions and edits to our work.

**Response to Additional Editor Comments**

We sincerely thank you for your thoughtful feedback and for recognizing the methodological rigor and coherence of our manuscript. We are also grateful for your constructive suggestions, which have been invaluable in refining the quality and impact of our work.

Below we outline our responses and corresponding revisions:

**Query 1:**

**Results Interpretation:** The regression outputs are clearly presented; however, expanding the interpretation to highlight the practical implications of significant predictors such as age, profession, and education on KAP outcomes would add depth and clarity.

**Response:**

We appreciate the suggestion to expand the interpretation of regression findings. In the revised manuscript, we have elaborated on the practical implications of significant predictors such as **age, profession, and education** in shaping knowledge, attitudes, and practices toward GDMT. These interpretations now better illustrate how specific demographic and professional characteristics influence clinical decision-making and guideline adherence (see Results*,* page 30, line # 484-499)

**Query 2:**

**Discussion**: While the study presents valuable findings, the discussion could benefit from a more robust comparison with regional or international GDMT implementation studies. This would help contextualize the results and improve their external relevance.

**Response**:

We have strengthened the discussion by comparing our findings with **regional and international GDMT implementation studies**, particularly those from South and Southeast Asia, Europe, and the Middle East. This addition contextualizes our results within a broader global framework and highlights shared challenges and opportunities for improvement in GDMT uptake (see Discussion*,* pages 31-33, line # 508-553).

**Query 3:**

**Limitations:** The manuscript appropriately acknowledges sampling constraints. Nonetheless, including a brief note on potential response bias or social desirability bias in self-reported practices would further enhance transparency.

**Response:**

We have expanded the Limitations section to acknowledge the potential for **response bias and social desirability bias**, given the self-reported nature of the KAP data. This addition improves transparency and helps readers interpret the findings with **appropriate caution (**see Limitations*,* page 35-36, line # 618-620).

**Query 4:**

**Conclusion:** The conclusion could be strengthened by offering more specific, actionable recommendations for integrating GDMT adherence training into multidisciplinary clinical settings. Emphasizing the evolving role of pharmacists, particularly in LMIC contexts, would underscore the study’s practical relevance.

**Response:**

We have revised the Conclusion to include **specific, actionable recommendations** for integrating GDMT adherence training within multidisciplinary healthcare teams. Additionally, we emphasized the **emerging role of pharmacists in LMIC contexts**, underscoring the importance of educational interventions and system-level integration to improve GDMT practice (see Conclusion*,* page 36, line # 626-633).

**Reviewer #2**

The manuscript has significantly improved, including the statement of the literature on the proposed subject matter, articulating the research question and alignment with the proposed aims/objectives, and rigorous methods that have been applied. Ample discussion was provided to substantiate the results and incorporation of additional literature which suggests due diligence on the part of the authors to conduct a comprehensive review on the subject matter.

**Response:**

We sincerely thank Reviewer #2 for their encouraging and constructive feedback. We are grateful for your recognition of the manuscript’s improvement, particularly regarding the expanded literature review, the clear articulation of research questions and objectives, and the methodological rigor applied throughout the study. Your positive evaluation affirms that the revisions have enhanced the manuscript’s clarity, coherence, and scientific quality.

We also appreciate your acknowledgment of the comprehensive discussion and incorporation of additional relevant literature. These revisions were undertaken with careful attention to your earlier suggestions and have substantially strengthened the contextual depth and interpretative value of our findings.

Thank you again for your thoughtful review and supportive comments. We believe the manuscript now provides a more complete and balanced contribution to the literature on GDMT knowledge, attitudes, and practices in low- and middle-income healthcare settings.

**Reviewer #3**

The manuscript demonstrates methodological rigor and strong alignment between research questions, hypotheses, and statistical analyses, but the discussion could further elaborate on how these findings compare with regional or international GDMT implementation studies to enhance external relevance.

While the results are clearly organized, the interpretation of regression outputs could be expanded to better illustrate the practical implications of significant predictors (e.g., age, profession, and education) on KAP outcomes.

The limitations section appropriately acknowledges sampling constraints; however, a brief note on potential response or social desirability bias in self-reported practices would improve transparency.
The conclusion could be strengthened by including more specific, actionable recommendations for integrating GDMT adherence training into multidisciplinary clinical settings, particularly highlighting pharmacists’ evolving roles in LMIC contexts.

**Response**

We sincerely thank Reviewer #3 for the constructive and detailed feedback, which has been instrumental in refining the manuscript.

**Discussion Expansion:** We have revised the Discussion to include comprehensive comparisons with recent regional and international GDMT implementation studies from South and Southeast Asia, Europe, and the Middle East (e.g., Zhang et al., 2024; Bekele et al., 2025; Schichtel et al., 2025). This addition contextualizes our findings within broader global patterns and enhances the manuscript’s external relevance. (see Discussion*,* pages 31-33, line # 508-553).

**Results Interpretation:** The Results and Discussion sections now provide an expanded interpretation of significant predictors (age, profession, and education), explaining their practical implications for GDMT-related knowledge, attitudes, and practice outcomes, with relevant literature support. see Results*,* page 30, line # 484-499)

**Limitations:** The Limitations section has been updated to acknowledge potential response and social desirability bias in self-reported practices, improving transparency regarding possible reporting effects. **(**see Limitations*,* page 35-36, line # 618-620).

**Conclusion:** The Conclusion has been strengthened to include specific, actionable recommendations such as integrating pharmacist-led multidisciplinary training programs, embedding GDMT adherence modules into continuing professional development, and emphasizing pharmacists’ evolving clinical roles in LMIC healthcare systems (see Conclusion*,* page 36, line # 626-633).

We appreciate the reviewer’s insightful suggestions, which have substantially improved the clarity, applicability, and scientific depth of the manuscript.
